# The Role of the Liver-Specific microRNA, miRNA-122 in the HCV Replication Cycle

**DOI:** 10.3390/ijms21165677

**Published:** 2020-08-07

**Authors:** Rasika D. Kunden, Juveriya Q. Khan, Sarah Ghezelbash, Joyce A. Wilson

**Affiliations:** Department of Biochemistry, Microbiology and Immunology, University of Saskatchewan, Saskatoon, SK S7N5E5, Canada; rasika.kunden@usask.ca (R.D.K.); jqk913@mail.usask.ca (J.Q.K.); sarah.ghezelbash@mail.mcgill.ca (S.G.)

**Keywords:** hepatitis C virus 1, microRNA 2, miR-122 3, replication 4, translation 5, RNA stabilization 6, tropism 7, pathogenesis 8

## Abstract

Hepatitis C virus (HCV) replication requires annealing of a liver specific microRNA, miR-122 to 2 sites on 5′ untranslated region (UTR). While, microRNAs downregulate gene expression by binding to the 3′ untranslated region of the target mRNA, in this case, the microRNA anneals to the 5′UTR of the viral genomes and upregulates the viral lifecycle. In this review, we explore the current understandings of the mechanisms by which miR-122 promotes the HCV lifecycle, and its contributions to pathogenesis. Annealing of miR-122 has been reported to (a) stimulate virus translation by promoting the formation of translationally active internal ribosome entry site (IRES) RNA structure, (b) stabilize the genome, and (c) induce viral genomic RNA replication. MiR-122 modulates lipid metabolism and suppresses tumor formation, and sequestration by HCV may influence virus pathogenesis. We also discuss the possible use of miR-122 as a biomarker for chronic hepatitis and as a therapeutic target. Finally, we discuss roles for miR-122 and other microRNAs in promoting other viruses.

## 1. Introduction

Hepatitis C virus (HCV) is thought to infect over 80 million people worldwide and can lead to serious liver problems, including cirrhosis and liver cancer [1]. It is transmitted through direct blood-to-blood contact and primarily via injection drug use [2,3] and 70% of infected individuals will develop a chronic liver infection. Many patients infected with HCV are unaware of their exposure because both acute and chronic infections are often asymptomatic [4]. However, unlike most other chronic viral infections, HCV can be cured. In the past, HCV was treated using a combination of pegylated-interferon and Ribavirin but the treatment had severe side effects and the rate of sustained virological response (SVR) was only about 50% [5]. In 2015, the release of interferon-free direct acting antiviral (DAA) therapy revolutionized HCV treatment and all DAA treatments now regularly achieve SVR rates to over 95% with few side effects [6]. However, identifying and treating individuals who do not know they are infected, poor adherence to therapy, and high therapy cost are barriers to worldwide elimination of HCV [7,8,9]. In addition, the high mutation rate of the virus may lead to the development of DAA resistance and a vaccine will be required to eliminate HCV infections worldwide [10]. Thus, molecular characterization of HCV infections is still required to develop novel HCV therapeutics and vaccines.

**Figure 1:**
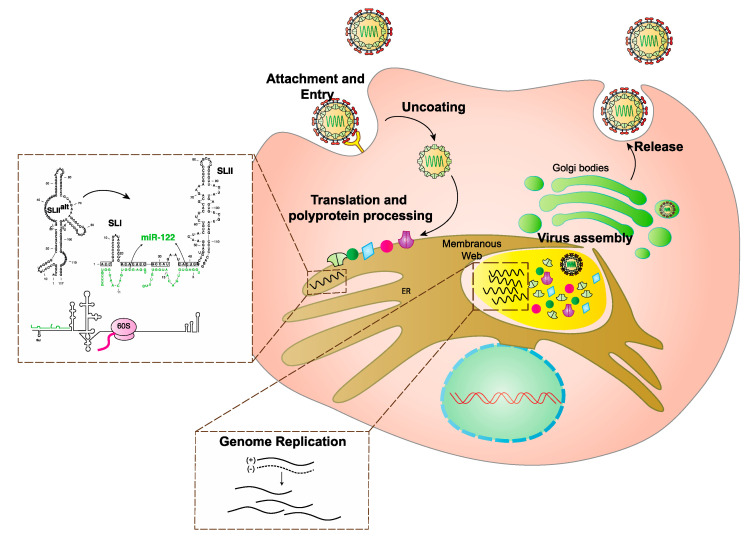
Schematic diagram of Hepatitis C Virus (HCV) life cycle. HCV binds to entry receptors and enters the cell by receptor mediated endocytosis. The virus then undergoes uncoating and direct translation. Translation takes place in association with the endoplasmic reticulum. Binding of miR-122 to the virus genome mediates an RNA structural change from the translationally inactive SLII^alt^ structure to translationally active IRES structure required to promote protein synthesis (process shown in dotted box). Viral proteins then stimulate formation of replication complexes where genome amplification (shown in dotted box) takes place. Assembly of the virion takes place in association with cellular membranes and the full formed virus is then released from the cell.

**Figure 2:**
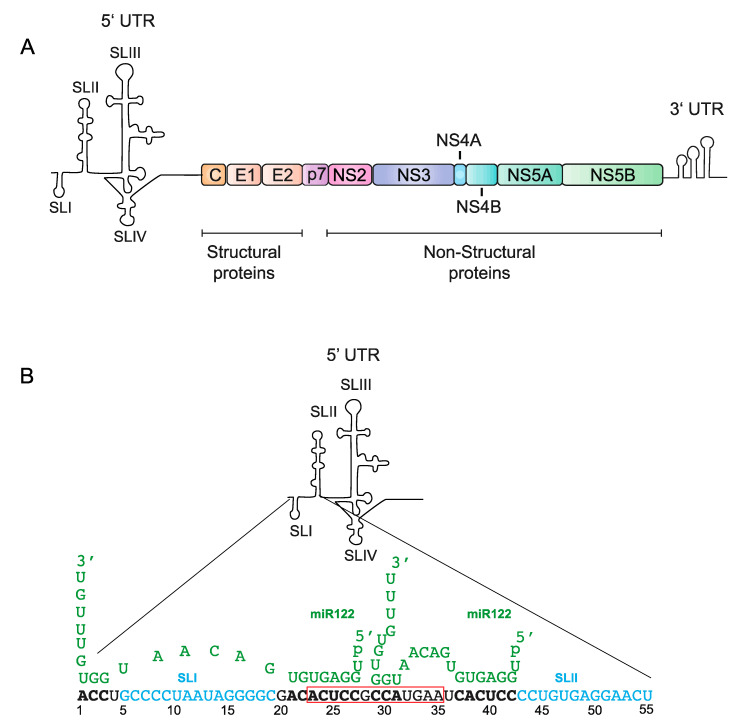
The HCV genome and interaction of its 5′untranslated region (UTR) with miR-122 (**A**) A schematic diagram of the genomic organization of HCV, which contains a polyprotein region of 10 genes, flanked by the highly conserved 3′ and 5′UTRs. (**B**) A diagram of the 5′UTR, including stem loops I-IV, as well as two copies of miR-122 (green) interacting with the first 55 nucleotides of the 5′UTR, with its binding nucleotides (bold). The red box indicates the minimum annealing region required for promotion by alternative small RNAs, as shown by Kunden et al. [21]. Mortimer et al. [22] and Chahal et al. [23] also showed potential interactions of these nucleotides with miR-122.

HCV is an enveloped positive (+) strand RNA virus belonging in the family Flaviviridae that primarily infects the liver [11]. The virus enters host cells using a variety of host cell surface factors [12], and immediately after virus entry, the genome is translated in association with host intracellular membranes (Figure 1). The virus generates a single polyprotein that is proteolytically processed into 10 viral proteins; viral non-structural proteins, NS3-NS5, are required for viral genome replication through generation of a minus strand intermediate [13]. NS2 and p7 are required for the assembly of viral particles and release of infectious virions but are not critical to RNA replication [14], and, viral structural proteins, core, E1, and E2 form HCV virions [15]. Like most + strand RNA viruses, translation, replication, and virion assembly are regulated by RNA elements formed by the 5′ and 3′ untranslated regions (UTRs) that fold into specific functional structures [16] (Figure 1 and Figure 2A). A unique aspect of HCV is its regulation by an RNA structure formed by annealing of two copies of a host liver specific microRNA, miR-122 to the 5′ terminus of its genome (Figure 2B). This is an unusual role for a miRNA since they normally suppress translation and destabilize mRNAs. The mechanism by which miR-122 promotes HCV is incompletely understood. In addition, unknown are the reasons the virus has evolved to rely on miR-122. HCV’s reliance on miR-122 has been speculated to affect viral pathogenesis by regulating virus tropism and perhaps by influencing the normal role of miR-122 in the cell [17,18]. Finally, miR-122 is a promising therapeutic target and sequestration of miR-122 by using a locked nucleic acid antagonist can reduce HCV RNA to undetectable levels in patients and provides promise for possible use in conjunction with DAA therapy [19]. However, recent evidence suggests the virus can develop resistance to miR-122-antagonist treatment [20]. In this review, we will provide an overview of the biogenesis and role of miR-122 in the liver, its role in HCV promotion and pathogenesis, and its promise as a therapeutic target. 

## 2. miRNA Biogenesis and the Cellular Functions of miR-122

Micro-RNAs (miRNAs) are small non-coding RNAs of approximately 22-nucleotides that silences gene expression by regulating mRNA translation and mRNA stability [24]. miRNAs are involved in nearly all developmental and pathological processes in animals and over 1000 different miRNAs have been identified in humans. miRNAs are transcribed endogenously by RNA polymerase II from host miRNA genes to generate primary miRNA (pri-miRNA). Pri-miRNAs are 5′ capped and 3′ polyadenylated RNA but instead of encoding proteins, form one or many hairpin stem-loop structures which are then cleaved by a microprocessor complex (Drosha and microprocessor complex subunit DCGR8) to form 70 to 100 bp precursor miRNAs (pre-miRNAs). The pre-miRNAs are subsequently transported from the nucleus to the cytoplasm, where they are processed by Dicer into miRNA duplexes of 18–25 nucleotides (nts). The miRNAs then associate with an Argonaute protein (Ago), within a larger protein complex called the RNA induced silencing complex (RISC) to form the miRISC. The miRNA duplex is unwound by Ago, releasing and discarding the passenger strand (sense strand), and the mature single-stranded guide strand directs the miRISC to partially complementary binding sites on the 3′UTR of mRNAs [25]. The principle determinant of miRNA target binding is the complementarity with the seed (nts 2–8), and to a lesser extent to auxilliary (nts 13–16) sequences of the miRNA. miRISC association results in mRNA translation suppression and degradation [26]. It is believed that at least one third of all human mRNAs are regulated by miRNAs. 

miR-122 is a liver specific miRNA. It is developmentally regulated, and its expression increases in the liver over the course of embryonic development [27]. It is present at approximately 66,000 copies per cell in adult liver, representing over 70% of the total liver miRNA pool, making it one of the most highly expressed miRNAs in any tissue [28]. miR-122 derives from a single genomic locus on chromosome 18 in humans and expression of miR-122 has been detected in 18 vertebrate species, including human, zebrafish, and frog [29]. The liver specific expression pattern and sequence of mature miR-122 is conserved from zebrafish to humans and no miR-122 orthologs have been detected in Drosophila or *C. elegans*. This indicates that miR-122 has evolved with the vertebrate lineage and possibly alongside the emergence of the liver. However, its role in the liver and significance of its abundance is incompletely understood.

Within the liver, miR-122 regulates cholesterol metabolism, iron homeostasis and is a tumor suppressor. The first miR-122 target RNA identified was Cationic amino acid transporter 1 (CAT-1) which mediates the Na^+^-independent transport of cationic amino acids. miR-122 binds to several sites in the CAT-1 3′UTR and mediates translational repression, accompanied by a shift of the repressed mRNA to P bodies [29]. The gross effect of miR-122 sequestration within the liver of mice [30,31] and non-human primates [31] is to lower the plasma cholesterol level. The pathways by which miR-122 targets play a role in modulating cholesterol metabolism are not fully understood, but it appears that indirect regulation of cholesterol biosynthesis genes may be important and confirmed mRNA targets include Aldolase A (AldoA) and N-myc downstream regulated gene 3 (Ndrg3) [31]. miR-122 has also been shown to modulate systemic iron homeostasis by supressing the target mRNAs, hemojuvelin (Hjv) and hemochromatosis (Hfe) [32]. These mRNAs encode activators of the hormone hepcidin, which regulates iron availability, and mice with reduced miR-122 levels suffer iron deficiency. Finally, miR-122 is a tumor suppressor and its expression is frequently reduced or abolished in hepatocellular carcinoma (HCC). Low expression or deletion of miR-122 in HCC has poor prognosis in cell lines [33] and in mice [27,34] and overexpression of miR-122 showed anti-tumorigenic properties of HCC in cell lines [35]. miR-122 overexpression also sensitizes HCC cells to chemotherapeutic agents like sorafenib and doxorubicin [36,37]. Further, multiple miR-122 target genes have been linked to inflammation, fibrosis, and tumorigenesis, including ADAM10, Igf1R, SRF, cyclin G1, and ADAM17 [38,39,40]. The factors governing reduced miR-122 expression in HCC have not been fully elucidated, but miR-122 levels correlate with those of several liver-specific transcription factors, including HNF-4a, suggesting a regulatory role for these proteins [41]. 

## 3. The Impact of miR-122 Annealing to the 5′UTR

An unusual role for miR-122 is its promotion of the replication cycle of HCV [42,43,44]. Annealing of two copies of miR-122 forms a trimolecular RNA structure that is essential for efficient virus propagation (Figure 2B) [22,23,43,45,46]. Similar to miRNA suppression, a major determinant of the efficiency by which miR-122 promotes HCV is its annealing pattern. Efficient HCV replication requires annealing of two copies of miR-122 and complementarity between HCV and the miR-122 seed site (nt 2–8), and the auxiliary site (nts 13–17). This leaves a loop of mismatched miRNA nucleotides between the seed and auxiliary sites and overhangs generated by its unannealed 5′ and 3′ miR-122 ends (Figure 2) [45,47]. Based on RNA SHAPE (Selective 2′-hydroxyl acylation analyzed by primer extension) and structure predictions, it was shown that the 3′ auxiliary binding of miR-122 at site 2 likely includes more extensive base pairing compared to auxiliary binding at site 1, and using isothermal titration colorimetry (ITC) miR-122 binding to site 2 had higher affinity than site 1 [22,23,45,46].

Although reports indicated the importance of the complex miR-122 binding pattern and the generation of a 7-nucleotide overhang for efficient HCV replication by miR-122, our group recently showed that this complexity is not required. We found that annealing of single perfectly matched small interfering RNAs (siRNAs) to the miR-122 binding region can also promote HCV replication as efficiently as 2 copies of miR-122 (as long as Ago2 mediated siRNA cleavage was abolished). We further mapped the locations on the HCV genome to which small RNA annealing induces HCV replication to nucleotides 1–3 and 15–44 and showed that annealing to nucleotide 45 and beyond do not. Further, siRNAs binding to 5′ terminus enhances virus replication but having a 3′ overhang did not, and actually supresses viral replication [21]. It therefore appears that any small RNA, binding within the boundary of nts 1–3 and 15–44, on HCV 5′UTR can promote the HCV lifecycle.

Several groups have also assessed the relative impact of miR-122 binding to each binding site on HCV replication promotion. Both miR-122 binding site 1 and site 2 are important, and efficient viral replication occurs only when both miR-122 binding sites are occupied [22,43]. The miRNA binds with higher affinity to site 2 than site 1 [22], and while some groups found that binding at site 1 was more important than binding at site 2 for replication promotion [48,49,50] others showed equal influences of each site [51]. We proposed neither site is more or less important, but that annealing to seed site 1 and accessory site 2 may be a key to efficient replication promotion. This is based on our recent finding that the siRNAs that promoted HCV replication most efficiently annealed to a small region (nts 23–35) that included these elements (Figure 2B, red boxed letters) [21]. That it takes annealing of two copies of miR-122 to anneal to these elements may explain why two copies of miR-122 is required and why annealing of miR-122 site 1 behaves similar to a conventional miRNA:target interaction, where binding to a seed site is important [22], and that miR-122 binding site 2 has higher affinity owing to extended base pairing at the accessory site. Thus, replication promotion appears to require annealing that includes the seed region of site 1 and the accessory region of site 2 [21]. 

## 4. The Role of other Predicted miR-122 Binding Sites on the HCV Genome

A bioinformatic search for miR-122 binding sites on the entire HCV genome reported 7 more predicted miR-122 binding sites. Four sites were predicted in the NS5B coding region and 3 more on the 3′UTR. One report suggested a negative role for miR-122 on a binding site in NS5B, while another report suggested that one binding site on the NS5B region is positively involved in regulating overall genome replication efficiency and a second binding site showed a weaker effect [52,53]. However, mutational studies performed to understand the involvement of these sites showed they did not affect HCV promotion and that the only functional miR-122 predicted binding sites were the two sites on 5′UTR [54]. Additionally, siRNAs annealing to these sites did not promote virus replication [21]. While a role for the alternative miR-122 binding sites remains unresolved, sequestration of miR-122 from target genes could be attributed to the multiple redundant binding locations.

## 5. The Mechanism of HCV Life-Cycle Promotion by miR-122

Three primary mechanisms by which miR-122 promotes virus replication have been reported, (a) stimulation of genome translation, and recent evidence suggests that miR-122 annealing may stimulate the generation of the canonical HCV IRES RNA structure, (b) genome stabilization, and (c) a direct role in promoting genome amplification. Since the HCV genome is a positive sense RNA, it is translated immediately after entry into the cell and, translation stimulation and genome stabilization would help initiate an HCV infection. Subsequent to translation, the viral non-structural proteins assemble into a replication complex that promotes viral RNA genome replication through generation of a negative strand intermediate and miR-122 has been proposed to directly promote genome amplification. In the following sections, we will provide details of the multiple mechanisms ascribed to miR-122, data supporting each mechanism, and the relative contributions of each mechanism on HCV RNA accumulation and the establishment of an HCV infection. 

### 5.1. miR-122 Stimulates HCV Translation

That miR-122 stimulates HCV translation was first reported in 2008 and has been confirmed by several groups [55]. miR-122 annealing stimulated translation from a reporter gene flanked by HCV 5′ and 3′UTR and from full length reporter HCV genome [21,46,55,56]. HCV translation is stimulated by annealing of miR-122 to either binding site 1 or 2. While the small 2-fold impact of miR-122 annealing on HCV translation had been considered in the past to be insufficient to account for its strong, 1000-fold increase in HCV replication, recent evidence supports an important role of translation stimulation by miR-122 on the HCV life cycle. Our group recently identified that siRNAs that anneal to the miR-122 annealing region promote HCV replication with different efficiencies and using these siRNAs we also found that their ability to promote replication correlates with their ability to promote translation [21]. This confirms a link between HCV replication promotion and translation stimulation and suggests that it is indeed a key pro-viral mechanism of miR-122. 

#### miR-122 Influences HCV Genomic RNA and IRES Structures

In addition, in support of an important role of miR-122 in promoting HCV translation, several recent studies have also proposed that miR-122 binding alters the structure of the HCV 5′UTR to promote and stabilize the canonical HCV IRES structure [43,44,45]. The HCV IRES is a structured RNA element formed by the HCV 5′UTR that regulates virus translation. The IRES structure and function has been analyzed in detail and defined to include several stem loop (SL) elements including (SLII, SLIII, SLIV), a pseudoknot, and 30 nucleotides within the core coding region [57,58]. The structure and functions of these elements have been well characterized [59,60,61]. SLII is divided into two parts, SLIIa which induces SLII to form a bent structure that directs SLIIb to the ribosomal E-site in the head region of the 40S subunit, facilitating 80S ribosome assembly [59,60,61].

The first 42 nucleotides on the 5′UTR are not considered part of the IRES and form SLI and an RNA structured element created by annealing of two copies of miR-122 [42,44,45,46]. Computational analyses of the 5′UTR RNAs that include SLI, the miR-122 annealing sites, and the IRES predict that the 5′UTR RNA will not form SLII, but will form a non-canonical structure termed SLII^alt^ [23,45,46]. SLII^alt^ is unlikely to support viral RNA translation but RNA structure predictions suggest that miR-122 annealing promotes formation of SLII and the canonical translationally active IRES structure [22,23,45,46]. Thus, this suggests a model in which miR-122 annealing induces the formation of the canonical HCV 5′UTR RNA IRES structure to promote virus translation and prevent alternative structure formations. However, that HCV translation is not abolished in the absence of miR-122 suggests that the 5′UTR RNA structure is dynamic, and that miR-122 annealing may shift the folding equilibrium toward that of the active HCV IRES RNA structure. Schult et al. [46]. proposed that the virus may have has retained this regulatory sequence through evolution might be due to the multifunctional nature of this region. In addition to regulating translation, the complementary sequences of the 5′UTR on 3′ end of the negative genomic strand forms RNA structures essential for genome amplification [62]. Thus, the virus may have evolved to use miR-122 annealing to prevent misfolding of the IRES caused by sequences required by the negative strand [21,23,46]. However, this model relies primarily on RNA structure prediction and has not been supported by direct biochemical or biophysical analyses.

**Figure 3:**
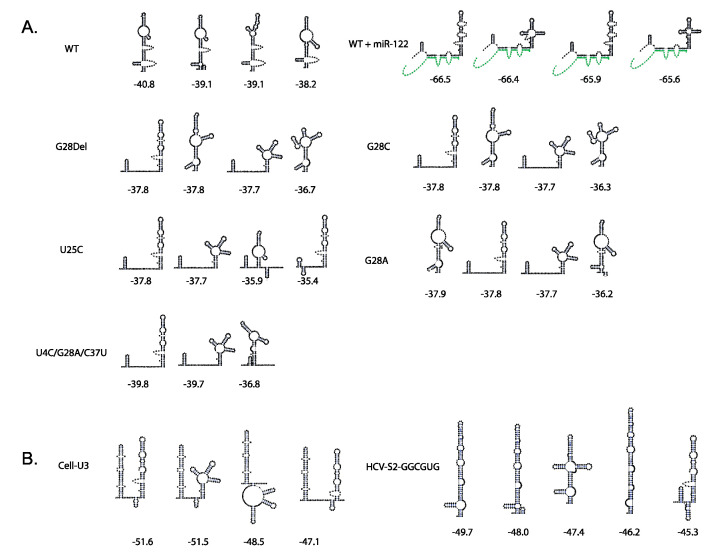
Secondary structure predictions of HCV mutants showing formation of SLII. (**A**) In silico secondary structure predictions (experimentally unvalidated structures) for the domain I and domain II of wild type HCV with and without miR-122 [21] and mutant RNA sequences of viruses capable of miR-122-independent replication [23,45,46]. One or more RNA structures show formation of translationally active IRES SLII structure for each mutant. (**B**) We performed secondary structure predictions using an online software ‘RNAstructure’ of Cell-U3 [50] and HCV-S2 GGCGUG [66] RNA sequences. These predicted structures showed formation of SLII in one or more structures which may be due to formation of another short stem loop upstream of SLII.

Indirect support for this model is provided by RNA structures predicted to be formed by mutant viruses that replicate independently of miR-122, and induced by siRNAs that promote HCV replication. Structure prediction programs, like RNAfold and RNAstructure, predict that the siRNAs that promote virus replication also induce the translationally active 5′UTR structure and those that do not promote HCV replication do not [45]. We also showed that annealing to nucleotides 23-35 is the minimum required for promotion of viral lifecycle and we speculate that annealing to this location may be optimal for inducing of the translationally active IRES structure since it resides within both non-base-paired regions to provide access to annealing, and base-paired regions so that its annealing can modulate the SLII^alt^ structure [21]. Further support for this model is derived from HCV genomes capable of miR-122-independent replication. Replication of full-length HCV RNA is undetectable in the absence of miR-122, even using sensitive luciferase-based reporter genomes, which suggests an essential (or near essential) role of miR-122 in HCV infections. However, there is now an accumulation of miR-122-independent HCV replication models. Some of the earliest models of miR-122-independent replication included dicistronic subgenomic HCV replicons (SGR) [63]. SGR RNA was found to replicate, albeit at lower levels, in a human hepatocyte cell line that lacks miR-122 expression (Hep3B). This finding supports the notion that miR-122 regulates translation since in the SGR, viral protein translation is regulated by an EMCV IRES instead of the HCV IRES, and thus may escape the need for miR-122 [63]. In addition, several full-length genomic mutants have been identified to support miR-122-independent HCV replication. Full length viruses having 5′ mutations U25C, G28A, and the combination mutations U4C, G28A, and U34G, were selected based on replication in miR-122-knockout cells, and in another report, several 5′UTR point mutations were found to support miR-122-independent replication [45,64,65]. Interestingly, most of the 5′UTR sequences that support miR-122-independent replication are also predicted to form the canonical 5′UTR SLII structure even in the absence of miR-122, suggesting that these viruses may lack the need for miR-122 because of mutation induced RNA structural changes (Figure 3A).

However, other full-length mutants that replicate independently from miR-122, Cell-U3, and HCV-S2-GGCGUG are not predicted to form the translationally active 5′UTR IRES structure and thus suggest the mechanism of replication promotion may be more complex. Cell-U3, a virus which contains a snoRNA in place of stem loop I and miR-122 binding site 1, was acquired from passage of SLI deleted viral genomes, and HCV-S2-GGCGUG was selected from a population of viruses having all possible sequences at miR-122 seed binding site 2 (Figure 3B) [50,66]. The RNA structures predicted to form by these viruses are shown in Figure 3B and were determined using the online software ‘RNAstructure’. The lowest free energy structures formed by these mutants does not include canonical SLII. However, we speculate that these RNAs may form structures that support efficient IRES translation, perhaps by forming hairpins within the miR-122-binding site region that stabilize SLII (Figure 3B). Confirmation of the role of miR-122 will require RNA structure and function analysis of mutant viruses and miR-122 annealing using in silico modelling combined with biochemical and biophysical methods.

### 5.2. MiR-122 Stabilizes the HCV Genomic RNA

miR-122 annealing also stabilizes the viral genome. The HCV genome has an uncapped 5′ end and it was hypothesized that 5′ terminus binding by miR-122 acts as an artificial cap to protect the triphosphate 5′ end from degradation by cellular phosphatases and 5′ exonucleases [47]. This hypothesis was supported by experiments showing that viral RNA was degraded by Xrn1 and to a smaller extent Xrn2, and that viral RNA degradation was slowed by annealing of miR-122 [51,67,68]. In addition, depletion of Xrn1 or Xrn2 partially restored HCV RNA accumulation when miR-122 was sequestered [51]. Recent advances further define miR-122 functions and show that miR-122 also protects the HCV genome from 5′ phosphatases Dom3Z and Dusp11, but not from innate sensors of 5′ triphosphate RNA ends [69,70]. However, depletion of Xrn1, Dom3z, and Dusp11 together did not restore wild-type replication of HCV in cells lacking miR-122 activity, indicating that protection from the cellular 5′ exonucleases Xrn1, Dom3z, and Dusp11 is not the only mechanism by which miR-122 promotes virus replication [69]. In addition, stabilization by small RNA annealing to the 5′UTR was not sufficient to induce detectable HCV replication since some siRNAs that could stabilize the viral genome did not promote virus replication [21]. Based on these data, we propose that genome stabilization enhances viral accumulation induced by miR-122 promotion of translation.

### 5.3. A Direct Role for miR-122 in Promoting Genome Amplification

It has been hypothesized that miR-122 has a direct effect on genome amplification and may act to regulate the switch from genome translation to genome replication. Using metabolic labeling and qRT-PCR to quantify RNA accumulation Masaki et al. [71] found that there was an increase in nascent viral RNA synthesis within 1 hr in cells transfected with miR-122, whereas there was no measurable increase in protein synthesis at that time, suggesting that miR-122 promotes genome replication before translation stimulation. Further, polysome profiling experiments showed that miR-122 promotes the transition from genomes undergoing translation to genome replication [65,71]. Finally, others hypothesized that miR-122 may promote positive-strand RNA synthesis by increasing the accessibility of the 3′ end of the template negative RNA strand [71,72]. However, the impact of miR-122 during ongoing virus replication is small, and because stable cells supporting miR-122-independent replication can be isolated, a direct role for miR-122 in replication promotion, it is not essential. Thus, the direct impact of miR-122 on the process of viral genome replication remains to be confirmed.

### 5.4. The Relative Contributions of each Mechanism to Promotion of the HCV Lifecycle

Understanding the mechanisms by which miR-122 promotes the HCV lifecycle has been a challenge and is still under debate. Thus far, efforts have determined roles for miR-122 in the HCV RNA stability, translation, and perhaps genome replication, but the relative impacts of each of these mechanisms on HCV lifecycle promotion are unknown. An emerging model that miR-122 annealing modifies the HCV RNA structure to activate the HCV IRES is intriguing but has not been confirmed biophysically. In addition, a link between the low levels of translation stimulation induced by miR-122 (2-4 fold) and the dramatic effect on viral RNA accumulation (1000 fold) remains a question, and may indicate that the relatively low threshold amount of translation stimulation is sufficient for efficient HCV replication. It also appears that miR-122 induced genome stabilization alone is not sufficient to promote the HCV lifecycle so may only enhance the virus lifecycle after induction of translation. Finally, a direct role in promoting viral genome replication independent from viral translation requires further confirmation.

## 6. Host Proteins Involved in miR-122 Promotion of the HCV Life Cycle

### 6.1. The Role of Ago2 in Genome Stabilization and Translation

The host protein Ago2 plays a role in miR-122 promotion of HCV, but its exact role is unknown. Ago1 and 2 interact with the HCV 5′UTR in association with miR-122 [73], and an Ago2 high-throughput sequencing and crosslinking immunoprecipitation (HITS-CLIP) study suggests that Ago interacts with the HCV 5′UTR at the two miR-122 binding sites, but also with regions in the HCV IRES whose sequences do not match known human miRNAs [17]. In addition, while a dominant role for Ago2 has been reported, evidence indicates other Ago isoforms (Ago1, 3, and 4) might also be involved [21,23,45]. Two hAgo2:miR-122 complexes are able to bind to the HCV 5′ terminus simultaneously and SHAPE analyses revealed further alterations to the structure of the 5′UTR to accommodate these complexes, suggesting that Ago2 may play a role in miR-122-induced RNA structure changes. Furthermore, computational models suggest that the hAgo2:miR-122:HCV RNA complex interacts with the IRES–40S complex in association with the HCV IRES, and that hAgo2 is likely to form additional interactions with SLII to further stabilize or activate the HCV IRES [23]. Perhaps annealing at position 23–35, the optimal nucleotides to which an siRNA can anneal and promote small RNA dependent HCV replication, is the best location to position Ago for this interaction [21]. Biophysical analyses of RNA-Ago complexes induced by annealing of siRNAs that promote the HCV lifecycle with varying efficiencies could clarify the molecular details of pro-viral nucleoprotein structures. HCV genome stabilization has also been proposed to be occurring due to recruitment of the Ago proteins by miR-122 on the 5′ terminus. Taken together, these reports support a new mechanistic model where miR-122 and Ago modify the 5′UTR RNA structure to stabilize the genome and induce viral IRES formation and translation stimulation.

### 6.2. Other Host Proteins Required for the Pro-Viral Activity of miR-122

Protein interactions with the 5′UTR in conjunction with or displaced by miR-122 are also speculated to mediate miR-122 promotion of HCV replication, but only few details are known. Apart from Ago, numerous host RNA binding proteins interact with the HCV genome and are proposed and shown to promote its replication. Those that bind the 5′UTR include La protein (SSB) [74], poly(rC)-binding protein 2 (PCBP2) [75,76], polypyrimidine tract-binding protein 1 (PTBP1), PTBP2 [77,78], heterogeneous nuclear ribonucleoprotein L (HNRNPL) [79], interleukin enhancer-binding factor 3 (ILF3; also known as NF90, NFAT90, NFAR) [80,81], insulin-like growth factor 2 mRNA binding protein 1 (IGF2BP1; also known as IMP1) [82], and the U6 snRNA-associated Sm-like (LSM) proteins LSM1–LSM7 [83], whereas synaptotagmin-binding cytoplasmic RNA-interacting protein (SYNCRIP; also known as HNRNPQ or NSAP1) binds to an RNA sequence immediately downstream of 5′UTR [84,85].

Several factors have been identified previously that regulate translation and circularization of the HCV genome. Proteins other than those regularly required in mRNA translation known as IRES- trans-activating factors (ITAFs) are identified. Interestingly, some ITAFs are shown to act as chaperons like the La protein and IMP1 protein, or even have roles in altering RNA structure like DDX6 [74,86]. Many cellular RNA-binding proteins have also been predicted to stimulate HCV genome circularization. PCBP2 has been identified to bind to the polio virus genome and has a role in its genome circularization and a similar role has been hypothesized for circularization of the HCV genome [75]. IGF2BP1 binds to the HCV 5′UTR and the 3′UTR and stimulates HCV genome translation and may also be involved in genome circularization [82]. Similarly, the cellular RNA chaperones NF90/NF45 were also shown to bind to the ends of the HCV RNA genome to promote virus replication and were predicted to facilitate genome circularization [87]. However, the crosstalk between these proteins and miR-122 if any, and their mechanism of HCV promotion are yet to be defined. It will be interesting to determine if any of these proteins are recruited to or displaced from the viral genome by miR-122 or due to the viral genome structural changes brought about by miR-122.

## 7. miR-122 Regulation of Virus Tropism and Pathogenesis

Why HCV has evolved to rely on miR-122 is unknown, but it is clear that miR-122 is essential for HCV replication in cell culture and miR-122 abundance in the liver undoubtedly regulates HCV tropism to the liver, and restriction of HCV replication to the liver likely benefits the virus [18]. The normal function of miR-122 is to regulate lipid and cholesterol metabolism, and the cellular conditions promoted by miR-122 might be advantageous for the virus. The liver is also suggested to be an immune-privileged environment and a reliance on miR-122 may also effectively silence HCV in non-hepatocyte cells and allows the virus to escape immune surveillance [88]. Evidence suggests evolutionary pressure to maintain a reliance on miR-122. Several mutant forms of HCV have been identified to replicate independent from miR-122 and based on error-prone virus replication, these mutations likely happen regularly during an HCV infection [45,50,65,66,89]. However, none of the mutants, except G28A, are observed in naturally occurring HCV sequences and suggests ongoing selection against miR-122-independent HCV replication during human infections [90]. However, experimental and clinical evidence suggests HCV can enter extrahepatic cells and HCV sequences can be amplified from peripheral blood mononuclear cells (PBMCs), B and T cells, monocytes/macrophages, dendritic cells, and other extrahepatic tissues of infected individuals, but whether HCV successfully replicates in extra-hepatic tissues is unresolved [91,92]. Serious complications of HCV infection include B-lymphocyte proliferative disorders, including mixed cryoglobulinemia and B-cell non-Hodgkin’s lymphoma, and one theory posits they are mediated by HCV infection of B cells [93,94,95]. While a reliance on miR-122 would limit HCV protein expression and abolish virus replication in these cells, it is unknown if miR-122 might be transported to these tissues via exosomes, or whether G28A viruses can replicate in these tissues [91]. Exosomes function as chemical messengers to mediate miRNA transport and cell–cell communication [96], and chronic liver damage results in release of miR-122 into the blood stream, suggesting that circulating miR-122 could facilitate HCV replication in extrahepatic tissues [97]. However, HCV infection of extrahepatic tissues and links with HCV disease complications remain controversial.

## 8. HCV as a miR-122 Sponge

Since HCV replication depends on miR-122, it was proposed that having an HCV infection would deprive the cell of miR-122 molecules required for its normal functions by sponging miR-122 within the cell. Using AGO HITS-CLIP and mRNA sequencing in HCV-infected and naïve cells, this was found to be true [17]. A significant global reduction in Ago binding and de-repression of endogenous miR-122 target mRNAs was observed. De-repression of miR-122 target genes was also stoichiometric, governed by both mRNA expression level and the number of miR-122 sites. These effects mimicked those observed during sequestration of miR-122 using antisense-locked nucleic acid inhibitors [17]. The miR-122 sponge effect was also observed in vivo based on analyses of liver biopsies of HCV-infected and naïve patients where miR-122 target genes were significantly de-repressed [17].

## 9. miR-122 as a Therapeutic Target and Viral Resistance to miR-122 Antagonisms

In view of the dependence of HCV on miR-122, miR-122 is a promising therapeutic target [19]. In phase I and II clinical trials, a miR-122 locked nucleic acid antagonist, miravirsen, induced a dose-dependent and sustained reduction in HCV viral loads up to 3 logs in the highest dose group, with several patients transiently achieving undetectable HCV RNA levels during the course of the study [98]. Miravirsen also acts additively when combined with direct-acting antivirals and thus, may be valuable as part of the drug cocktail to treat patients who do not respond to direct-acting antivirals alone [99]. However, all subjects experienced a virologic rebound after several weeks of treatment [100] and long-term therapy has not been tested due to caution regarding inhibiting a known tumor suppressor. Sequence analysis of viral RNAs obtained from serum of several of the patients treated with miR-122 antagonists suggest the emergence of resistance. Sequencing revealed a resistance-associated substitution of a uridine for a cytidine nucleotide 3 (C3U) [101]. This mutation occurred in the auxiliary region of miR-122 binding site 1 and biochemical assays showed reduced binding of miR-122 at site 1 and destabilization of the tri-molecular structure formed by 2 copies of miR-122 and the HCV RNA. However, this mutant displayed higher rates of replication when miR-122 abundance was low, suggesting miR-122 independent replication. Interestingly, the C3U mutant has not been observed in cell culture selection and has only been observed after several weeks of anti-miR122 treatment in patient serum and only transiently [20,65,90]. Further, since there is no evidence of presence of C3U in infected livers of patients, this suggests that C3U may only live transiently in hepatocytes or may occur due to extra hepatic infections. The location of this mutation suggests that it may affect genome stability and not IRES structure and function. Because evidence suggests that stabilization of the HCV genome is not sufficient to rescue HCV replication in absence of miR-122, then this virus may not be viable in cell culture or to initiate a new infection. However, this may explain why it has never been observed in cell culture selections. Further, Israelow et al. [90] first described that an HCV variant, G28A, can replicate in absence of miR-122 and another group demonstrated that after serial passages of HCV in Huh7.5.1 miR-122KO cells, G28A adaptive mutation was observed [65]. Competition assays revealed that the G28A mutation does not confer an advantage for propagation in miR-122 rich hepatocytes but was also isolated from PBMCs of HCV infected patients. This suggests that G28A is maintained in hepatocytes and may be selected dominantly in PBMCs. These findings indicate that the occurrence of HCV mutants that can grow in non-hepatic cells in a miR-122-independent manner may induce resistance to miR-122-antagonist therapy.

## 10. Circulating miR-122 as a Biomarker for Chronic Viral Hepatitis Detection

Circulating miRNAs are secreted from cells via exosomes and micro vesicles during the process of cell death. Such secreted miRNAs can be stably detected from body fluids like serum, plasma, urine, and cerebrospinal fluid (CSF), making them relatively non-invasive biomarkers to detect infectious diseases in patients [102,103]. As a major miRNA in the liver, miR-122 has been widely reported to suffer from dysregulation in HCV and hepatitis B virus (HBV) infection. The diagnostic value of circulating miR-122 in chronic viral hepatitis was verified and assessed by a systematic literature review and conducting a meta-analysis [104]. Fifteen studies were included in their meta-analysis according to the exclusion and inclusion criteria, and the subgroup analysis demonstrated that the level of miR-122 correlated with the severity and stage of infection and helped in evaluating the treatment responses. It was observed that, the diagnostic accuracy was better for HCV-associated chronic hepatitis patients and non-Chinese compared with other subgroups. The authors further found that serum might be a more promising matrix for detecting the expression of miR-122 than plasma. Thus, they conclude that the circulating miRNA-122 has a relatively high diagnostic value for chronic viral hepatitis detection, especially in patients of HCV-associated chronic hepatitis [104]. In the future, it will be beneficial to consolidate these results by performing well-designed, large-scale meta-analysis in clinical practice.

## 11. Interaction of miR-122 with other Viruses of the Genus *Hepacivirus*

While initially considered a unique phenomenon to HCV, recent discoveries have highlighted the interaction of miR-122 with other viruses within the genus *Hepacivirus*. In most other viruses, the miR-122 dependency for replication has been maintained such as in Norway rat hepacivirus (NrHV) or rodent hepacivirus-nr-1 (RHV-nr-1), which can establish a hepatotropic infection in rats only in presence of miR-122 [105]. Bovine hepacivirus (BovHepV) and equine nonprimate hepacivirus (NPHV) are other viruses found to depend on miR-122 for translation [106,107]. This could be attributed to the structural similarities of the 5′UTR and IRES between HCV and other *hepaciviruses*. However, the miR-122 binding sites and locations differ.

**Figure 4:**
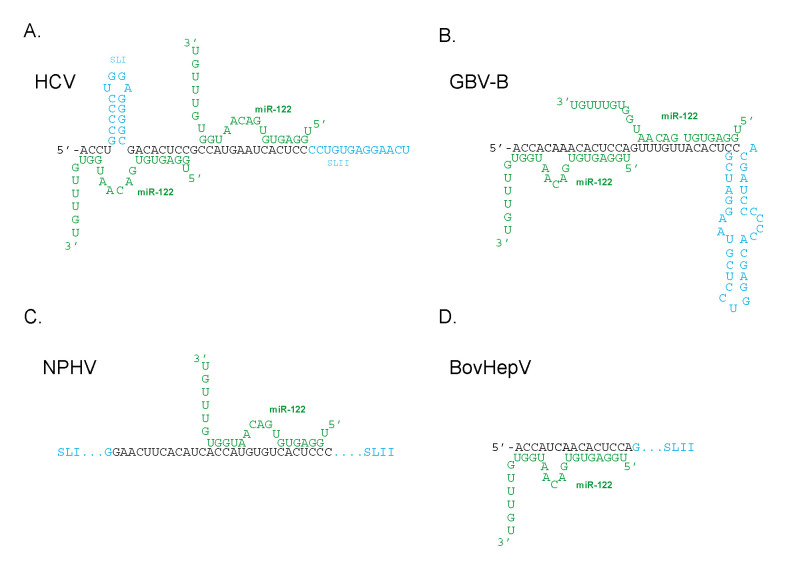
Schematic diagram of miR-122 binding sites on the 5′UTR of other *Hepacivirus*. MiR-122 (shown in green) interacting with 5′UTR of (**A**) hepatitis C virus, (**B**) GB virus B, (**C**) equine nonprimate hepacivirus, (**D**) bovine hepacivirus. SLI and SLII denote stem loop 1 and 2 respectively on the 5′UTR

The 5′UTR of RHV-nr-1 is structurally similar to the HCV 5′UTR containing two miR-122 seed sites, with the first miR-122 seed site being more important than the second [108]. NPHV, considered to be the closest relative of HCV and while was originally thought to have two miR-122 seed sites, was found to bind a single miR-122 to a site that corresponds to the second miR-122 binding site in HCV (Figure 4) [66]. In contrast, BovHepV contains only one putative miR-122 binding site, corresponding to the first miR-122 binding site in HCV (Figure 4) [106]. GBV-B, the first known homolog of HCV, was also found to have two miR-122 binding sites (Figure 4) and virus accumulation depended on miR-122 and Ago2 abundance. However, a mutant lacking both the miR-122 binding sites was found to grow independently of miR-122 and was insensitive to miR-122 supplementation, miR-122 sequestration, and Ago2 depletion [109]. Thus, GBV-B appears to be able to simply delete the modulatory RNA to which miR-122 anneals to abolish the virus’ dependence on miR-122. HCV cannot delete the miR-122 annealing region because the 3′UTR complement of this region is essential for virus RNA replication [62], but that GBV-B can delete this region suggests that dependence of GBV-B on miR-122 did not evolve to compensate for essential minus strand sequences. Some level of miR-122 independent replication was also observed with NPHV IRES containing NPHV/ HCV chimera viruses [66]. These viruses provide alternative models for RNA structure and functional analyses to further define the impact miR-122 annealing has on viral genomes.

## 12. Promotion of other RNA Viruses by Annealing of Host miRNAs

**Figure 5:**
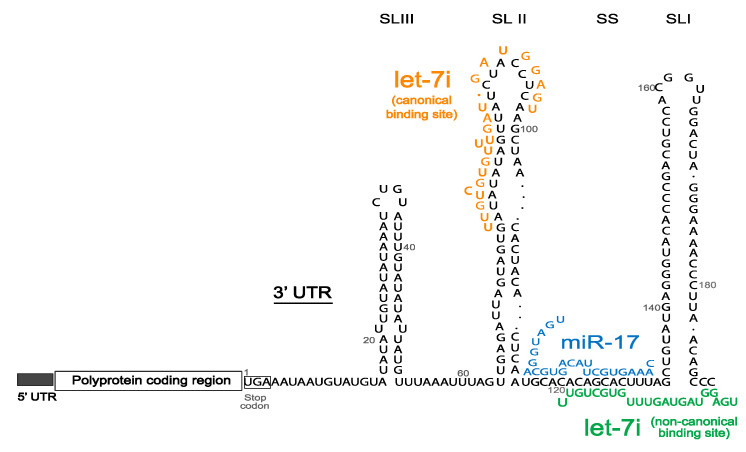
Schematic diagram of bovine viral diarrhea virus (BVDV) genome with 5′UTR, polyprotein coding region and the three stem loops (SLI-III) of 3′UTR, with binding of miR-17 (blue), and let-7 at canonical (orange) and non-canonical (green) sites.

In 2016, Scheel et al. [110] used crosslinking immunoprecipitation (CLIP) of the Ago proteins to identify virus-miRNA interactions for 15 different clinically relevant RNA viruses, leading to the discovery of the only other virus group, genus *Pestivirus,* so far known to depend on miRNA interactions for viral replication. Bovine viral diarrhea virus (BVDV) and classical swine fever virus (CSFV) are important animal pathogens belonging to the *Pestivirus* genus, within the *Flavivirdae* family [111]. The replication of these viruses critically depends on the interaction of the viral 3′UTR with two microRNA families, miR-17 and let-7, with their canonical binding regions playing a major role as compared to the non-canonical let-7 binding site (Figure 5) [110]. miR-17 and let-7 were also found to positively regulate BVDV translation, whereas miR-17 alone stabilized the BVDV genome [110]. A structural switch has been hypothesized to contribute to genome stability as well as enable regulation of virus translation and replication [112]. miRNA sponging effect, as seen with HCV and miR-122, was observed for miR-17 but not let-7 in BVDV and CSFV infected cells and Ago2 binding was implicated to be essential for BVDV [110,112]. These similarities suggest a mechanistic intersection between the two virus groups, despite the different annealing locations, the 5′UTR in the case of HCV versus the 3′UTR for BVDV. Studying the interactions of these viruses with miR-17 and let-7 miRNAs, structurally and functionally, will allow us to understand the role of small RNA annealing and RNA structures in the virus life cycle and can lead to development of additional models to study miRNA-virus interactions

## 13. Concluding Remarks

The reliance of HCV on the liver-specific microRNA, miR-122, evidently governs tissue tropism and provides glimpses into the evolution of HCV and related viruses. There is growing evidence that miR-122 annealing to the HCV genome influences the virus at several stages, including genome stabilization, translation, genome amplification, protein recruitment, and miR-122 sequestration, but the exact contributions at each of these steps to virus replication and pathogenesis is still unknown. Understanding the structural changes brought about by small RNA annealing to viral genome can lead us to understand the relationship between RNA structures and viral function as well as protein recruitment. In addition, the localization and temporal pattern of miR-122 and associated protein interactions during the virus life cycle will provide insight into the role of miR-122-viral genome interaction in establishing the infection and continuing ongoing replication. Assessing the impact of miR-122 and possible Ago2 sequestration on cellular gene targets will also allow us to identify impacts of HCV infection on host regulation and pathogenesis. Finally, studying other viruses that also interact with host microRNAs can provide mechanistic insight and a better understanding of how microRNAs affect positive-stranded RNA viruses.

## Abbreviations

Ago	Argonaute protein
Aldo	Aldolase A
BovHepV	Bovine hepacivirus
BVDV	Bovine viral diarrhea virus
CAT-1	Cationic amino acid transporter 1
CLIP	Crosslinking immunoprecipitation
CSF	Cerebrospinal fluid
CSFV	Classical swine fever virus
DAA	Direct acting antiviral
EMCV	Encephalomyocarditis virus
HBV	Hepatitis B virus
HCC	Hepatocellular carcinoma
HCV	Hepatitis C virus
Hfe	Hemochromatosis
HITS-CLIP	High-throughput sequencing and crosslinking immunoprecipitation
Hjv	Hemojuvelin
IRES	Internal ribosome entry site
ITAFs	IRES- trans-activating factors
ITC	Isothermal titration colorimetry
miR-122	microRNA-122
miRNAs	microRNAs
Ndrg3	N-myc downstream regulated gene 3
NPHV	Non-primate hepacivirus
NrHV	Rat hepacivirus
Nts	nucleotides
PBMCs	Peripheral blood mononuclear cells
PCBP2	Poly(rC)-binding protein 2
pri-miRNA	primary miRNA
PTBP1	Polypyrimidine tract-binding protein 1
RHV-nr-1	Rodent hepacivirus-nr-1
RISC	RNA induced silencing complex
SGR	Subgenomic HCV replicons
SHAPE	Selective 2′-hydroxyl acylation analyzed by primer extension
siRNAs	Small interfering RNAs
SL	Stem loop
SSB	La protein
SVR	Sustained virological response
UTR	Untranslated region
